# Aqueous Binders for Electrochemically Stable VOPO_4_ 2H_2_O Anodes for Li‐Ion Storage

**DOI:** 10.1002/open.202500102

**Published:** 2025-06-17

**Authors:** Alexander Beutl, Andrea Paolella, Yuri Surace, Qixiang Jiang, Marcus Jahn, Artur Tron

**Affiliations:** ^1^ Center for Transport Technologies Battery Technologies AIT Austrian Institute of Technology GmbH Giefinggasse 2 1210 Vienna Austria; ^2^ Dipartimento di Scienze Chimiche e Geologiche Università degli Studi di Modena e Reggio Emilia Via Campi 103 41125 Modena Italy; ^3^ Polymer and Composite Engineering (PaCE) Group Insitute of Materials Chemistry & Research Faculty of Chemistry University of Vienna Währinger Str. 42 1090 Vienna Austria

**Keywords:** hydrothermal method, lithium‐ion batteries, polyacrylic acid, polyvinylidene difluoride, sodium carboxymethylcellulose, VOPO_4_

## Abstract

The use of polyvinylidene difluoride (PVDF) binder dissolved in toxic N‐methyl pyrrolidone (NMP) organic solvent needs to be decreased during the manufacturing battery process to improve safety. Therefore, the development of aqueous‐based binders for negative and positive electrodes is necessary to make the production environmentally friendly. This study reports the performance of hydrothermal VOPO_4_ 2H_2_O anode material in combination with aqueous‐based binders such as sodium carboxymethylcellulose (CMC), polyacrylic acid (PAA) and their mixture CMC‐PAA (1:1 wt%). The aqueous binders are compared to standard PVDF as a reference binder. The cells with aqueous‐ and PVDF‐based electrodes are tested by galvanostatic, cyclic voltammetry, and electrochemical impedance spectroscopy measurements. When the CMC‐PAA aqueous binder is used, the electrode displays the most stable electrochemical performances due to a uniform distribution of the VOPO_4_ with strong adhesion to the current collector for long‐term cycle life. A stable, solid electrolyte interphase layer is formed when the mixture of CMC‐PAA is used instead of a standard PVDF‐based binder. In addition, these samples display stable cycling life at different rate capabilities due to the facilitated lithium‐ion diffusion and electronic conductivity.

## Graphical abstract

This study evaluates hydrothermal VOPO_4_·2H_2_O anodes with eco‐friendly aqueous binders— sodium carboxymethylcellulose (CMC), polyacrylic acid (PAA), and a CMC‐PAA blend—against conventional polyvinylidene difluoride. The CMC‐PAA binder offers superior adhesion, uniform electrode structure, and stable, solid electrolyte interphase formation, enhancing lithium‐ion diffusion and cycling stability. These findings support sustainable, high‐performance lithium‐ion battery development.

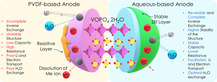

## 1 Introduction

Since their commercialization in the 1990s, lithium‐ion batteries represent a technology with a high impact on our modern life, with currently growing markets for small‐ and large‐scale applications.^[^ ^1^ ^]^ New materials are necessary to achieve fast charge–discharge systems, which will provide high safety and long cycle life for the batteries.^[^ ^2^, ^3^ ^]^ Anode and cathode materials should be able to deliver high specific capacities 120–200 mAh g^−1^ for the transition metal oxide cathode and 372 mAh g^−1^ for anode materials.^[^ ^4^, ^5^ ^]^

Among several anode candidates, the phosphate‐based electrode materials represent a valuable candidate due to the robust PO_4_^3−^ anion, which makes the battery with a) a high specific capacity, b) a high energy density, and c) an excellent rate capability as previously reported.^[^ ^5^, ^6^, ^7^, ^8^, ^9^ ^]^ VOPO_4_‐based material has also been studied as a cathode with various structures for postlithium‐ion batteries as well as magnesium‐ and sodium‐ion batteries.^[^ ^10^, ^11^, ^12^, ^13^, ^14^, ^15^ ^]^ It should be noted that the vanadyl phosphates material presents seven phases with different structures: β‐ and ε‐types have open tunnels, while the αl‐, αll‐, δ‐, ω‐, and γ‐phases have layered structures.^[^ ^10^ ^]^ Among them, thanks to the layered structure, αll‐type VOPO_4_ can provide high theoretical capacity (827 mAh g^−1^) due to good crystallinity and the combination of VO_6_ octahedral and PO_4_ tetrahedral units, resulting in a stable structure during the lithium ions intercalation/deintercalation processes.^[^ ^16^, ^17^ ^]^ In literature, VOPO_4_‐based electrodes were prepared by using polyvinylene difluoride (PVDF) binder. However, due to the F atoms on the linear chain structure, PVDF can form a LiF insulating layer, leading to capacity battery fade.^[^ ^16^, ^18^ ^]^ PVDF is solubilized in organic solvent N‐methyl pyrrolidone (NMP) with high safety risk due to NMP toxicity.^[^ ^19^, ^20^, ^21^, ^22^ ^]^ As shown in **Table** **1**, the various organic‐ and aqueous‐soluble binders were applied for the electrode formulation,^[^ ^22^, ^23^, ^24^, ^25^ ^]^ it should be noted that PVDF is a binder commonly used for electrode preparation due to the good binding ability to active material and the current collector wettability with reasonable electrochemical stability and maintaining facile lithium transport. However, due to the insufficient mechanical strength of PVDF, this binder is rarely used for high‐energy anode materials, which cannot support a large volume change during the cycling process.^[^ ^23^ ^]^ VOPO_4_ 2H_2_O anode material for lithium‐ion battery chemistries with aqueous‐based binders has been rarely investigated; in this work, hydrothermal VOPO_4_ 2H_2_O was investigated as anode material for lithium‐ion batteries and it was combined with different binders such as carboxymethyl cellulose (CMC), polyacrylic acid (PAA), and their mixture (CMC‐PAA 1:1, wt%) to standard PVDF binder. The final electrochemical performances indicate that the VOPO_4_ 2H_2_O anode with aqueous‐based binders shows stable specific capacities and lower surface resistance concerning electrodes made using PVDF binder. Furthermore, this study is attractive for designing future eco‐friendly and cost‐effective binders aimed at further enhancing the electrochemical performances of the active electrode materials.

**Table 1:** A summary of organic‐ (PVDF) and water‐soluble (CMC, PAA, and CMC+PAA) binders for the electrode with high energy density.

	Binder			
	PVDF	CMC	PAA	CMC+PAA
Chemical formula	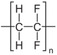	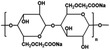	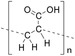	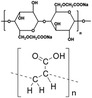
Polymer chemistry	Linear type (homopolymer)	Linear type (homopolymer)	Linear type (homopolymer)	Crosslinked (3D crosslinked structure based on chemical covalent bond)
Fluoride free	yes	no	no	no
Processability	Organic solvent‐based slurry (e.g., NMP)	Water/ethanol‐based slurry	Water/ethanol‐based slurry	Water/ethanol‐based slurry
Synthesis	Nonaqueous chemistry involving fluorinated chemicals	No chemistry involved (natural binder) or aqueous chemistry	No chemistry involved (natural binder) or aqueous chemistry	No chemistry involved (natural binder) or aqueous chemistry
Sustainability	Derived from nonrenewable sources	Obtainable from renewable sources	Obtainable from renewable sources	Obtainable from renewable sources
Mechanical properties	medium	medium, high	medium, high	medium, high
Easy of disposal	Hydrometallurgical or pyrometallurgical methods required and F emission	Recyclable by water treatment (simple washing	Recyclable by water treatment (simple washing	Recyclable by water treatment (simple washing
Green Score	low	high	high	high
Application	Cathode materials Unlimited anode materials	Si‐based anode LiCoO_2_ LiNi_0.1_Mn_1.5_O_4_ LiNi_1/3_Co_1/3_Mn_1/3_O_2_ S‐based electrode	Si‐based anode S based electrode	Si‐based anode LiCoO_2_ LiNi_0.1_Mn_1.5_O_4_ LiNi_1/3_Co_1/3_Mn_1/3_O_2_

## 2 Results and Discussion

**Figure 1:**
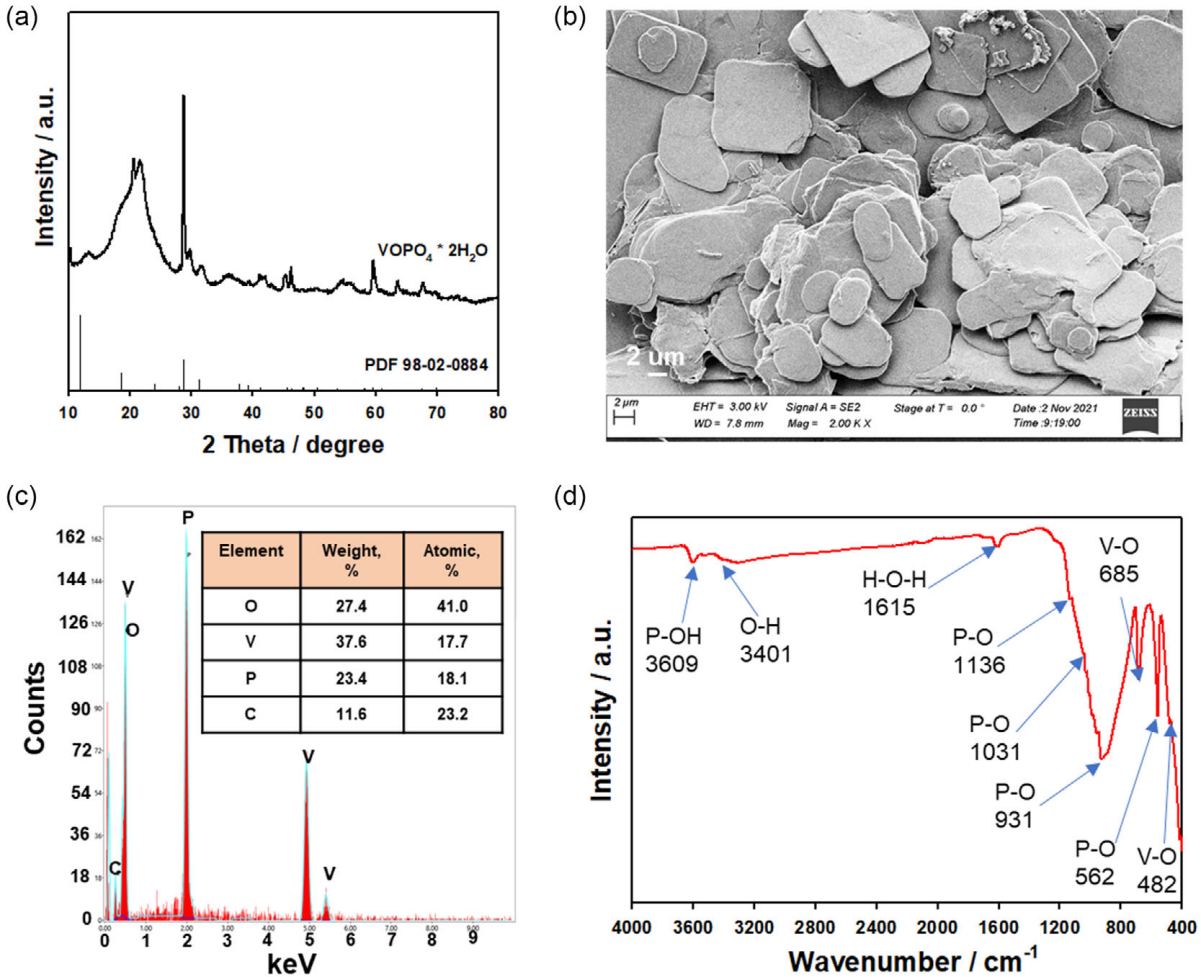
a) XRD pattern, b) SEM image, c) EDS analysis, and d) FTIR analysis of as‐synthesized VOPO_4_ 2H_2_O material.

**Figure 2:**
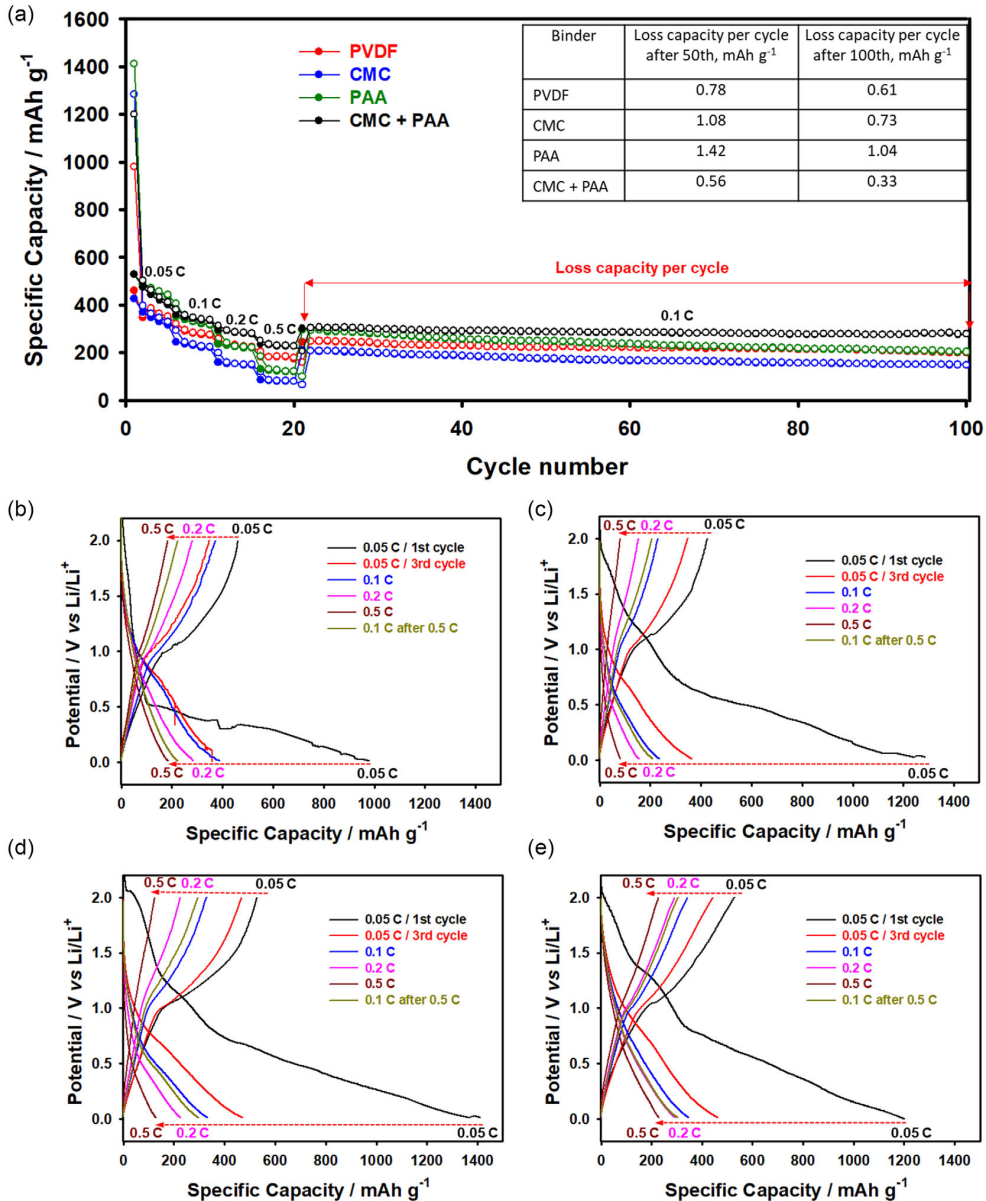
a) Rate capability with cycling life and voltage curves of cycling life from Figure 2a of VOPO_4_ 2H_2_O material in the organic electrolyte of 1 M LiPF_6_ in EC DEC (1:1, v/v) with b) PVDF, c) CMC, d) PAA, and e) CMC+PAA binders in the potential range of 0.01–2.0 V at 25 °C.

As‐synthesized VOPO_4_ 2H_2_O anode material was structurally characterized by X‐ray diffraction (XRD) analysis, as shown in **Figure** **1**a, the pattern shows a high crystallinity and structure without visible changes and impurities, and the presence of the alpha phase with a layered structure (PDF card number 98‐02‐0884).^[^ ^26^ ^]^ Moreover, thanks to the hydrothermal method which is used for the synthesis of the anode and cathode materials and also surface modification and/or coatings can reach a high crystallinity of structure, better distribution of particle size resulting in the stable electrochemical cycle life, and possible preparation of this material by the hydrothermal process in the large scale with lower cost effect.^[^ ^27^, ^28^ ^]^ The scanning electron microscopy (SEM) analysis confirms the formation of particles having a platelet shape with rough surfaces, as shown in Figure 1b. The energy‐dispersive X‐ray spectroscopy (EDS) results show a good elemental distribution of the V and P elements without any phase segregation as a consequence of side reactions (Figure 1c), and the obtained results are in agreement with inductively coupled plasma optical emission spectrometry (ICP‐OES) analysis (ratio V:P is 24.1:15:7 w/w%). It should be noted that the presence of residual carbon element on the VOPO_4_ particle surface is common for anode and cathode materials because of the inert synthetic conditions leading a precursor carbonization.^[^ ^6^ ^]^ Generally, the final amount of carbon does not have any impact and crucial contribution on the cycling data of VOPO_4_ particles during long‐cycle life. Additionally, the Fourier transform infrared spectroscopy (FTIR) analysis was applied to as‐synthesized VOPO_4_ 2H_2_O powder to confirm the existence of PO, VO, and HOH groups, as shown in Figure 1d, in agreement with previous references.^[^ ^29^, ^30^, ^31^ ^]^ The absorption peaks located at about 685 and 482 cm^−1^ are attributed to the V–O stretching vibration referred to the VO_6_ group. The peaks at 1136, 1031, 931, and 562 cm^−1^ correspond to the P–O stretching vibration in the PO_4_ group. The absorption peaks of P–OH and O–H stretching vibration in H_2_O are observed at 3609 and 3401 cm^−1^, respectively. The peak at 1615 cm^−1^ is related to the H–O–H bending vibration of H_2_O. The FTIR peaks are in agreement with the chemical composition of hydrothermal VOPO_4_ 2H_2_O powder. It should be noted that the obtained results confirmed the stable electrochemical performance, as shown in **Figure** **2** and Figure S1 (Supporting Information).

**Figure 3:**
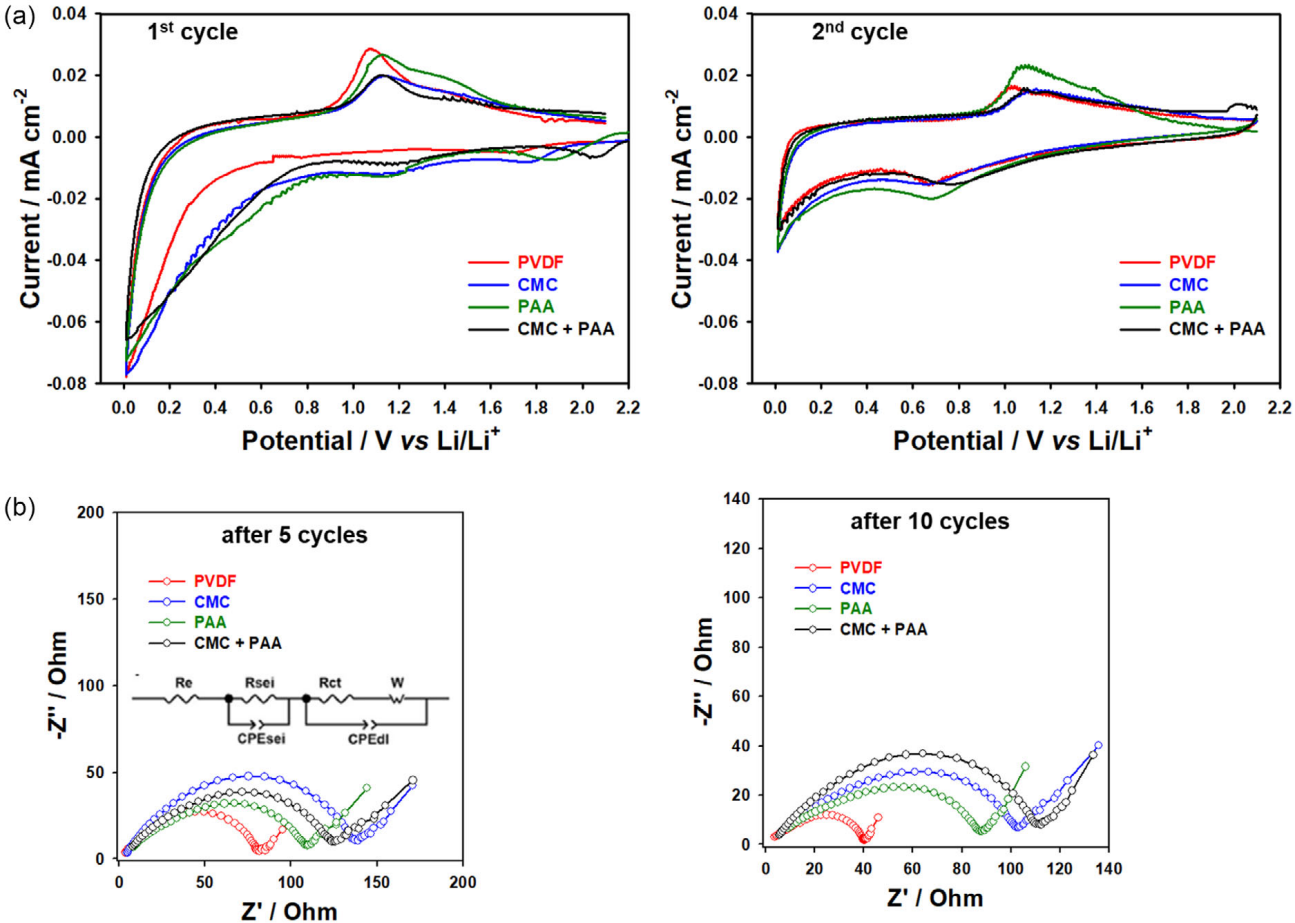
a) CV in the potential range of 0.01–2.1 V at a scan rate of 0.01 mV sec^−1^ of VOPO_4_ 2H_2_O material in the organic electrolyte of 1 M LiPF_6_ in EC DEC (1:1, v/v) with PVDF, CMC, PAA, and CMC+PAA binders, and b) Nyquist plots of VOPO_4_ 2H_2_O material in the potential range of 0.01–2.0 V at 25 °C for 100 cycles.

**Figure 4:**
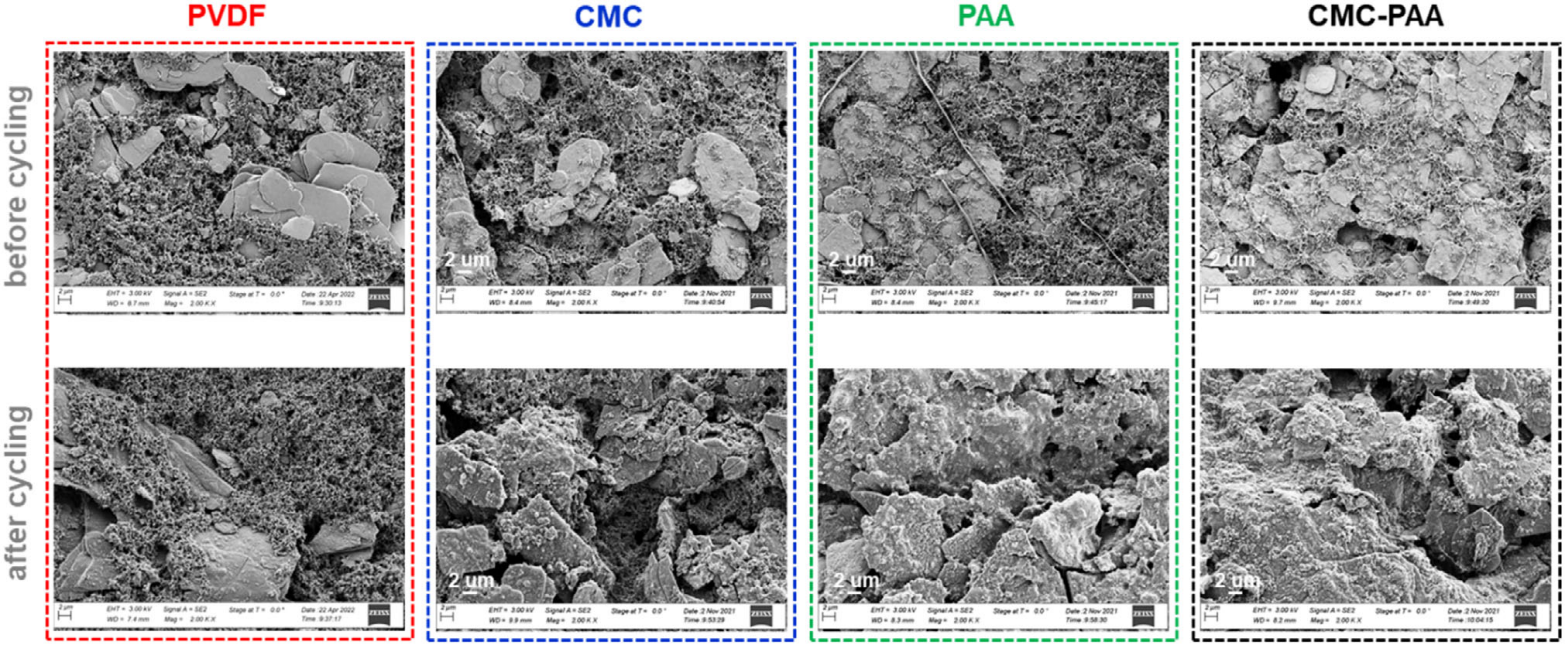
SEM analysis of VOPO_4_ 2H_2_O surface anodes before and after cycling in the organic electrolyte of 1 M LiPF_6_ in EC DEC (1:1, v/v) with PVDF, CMC, PAA, and CMC+PAA binders in the potential range of 0.01–2.0 V at 25 °C.

**Figure 5:**
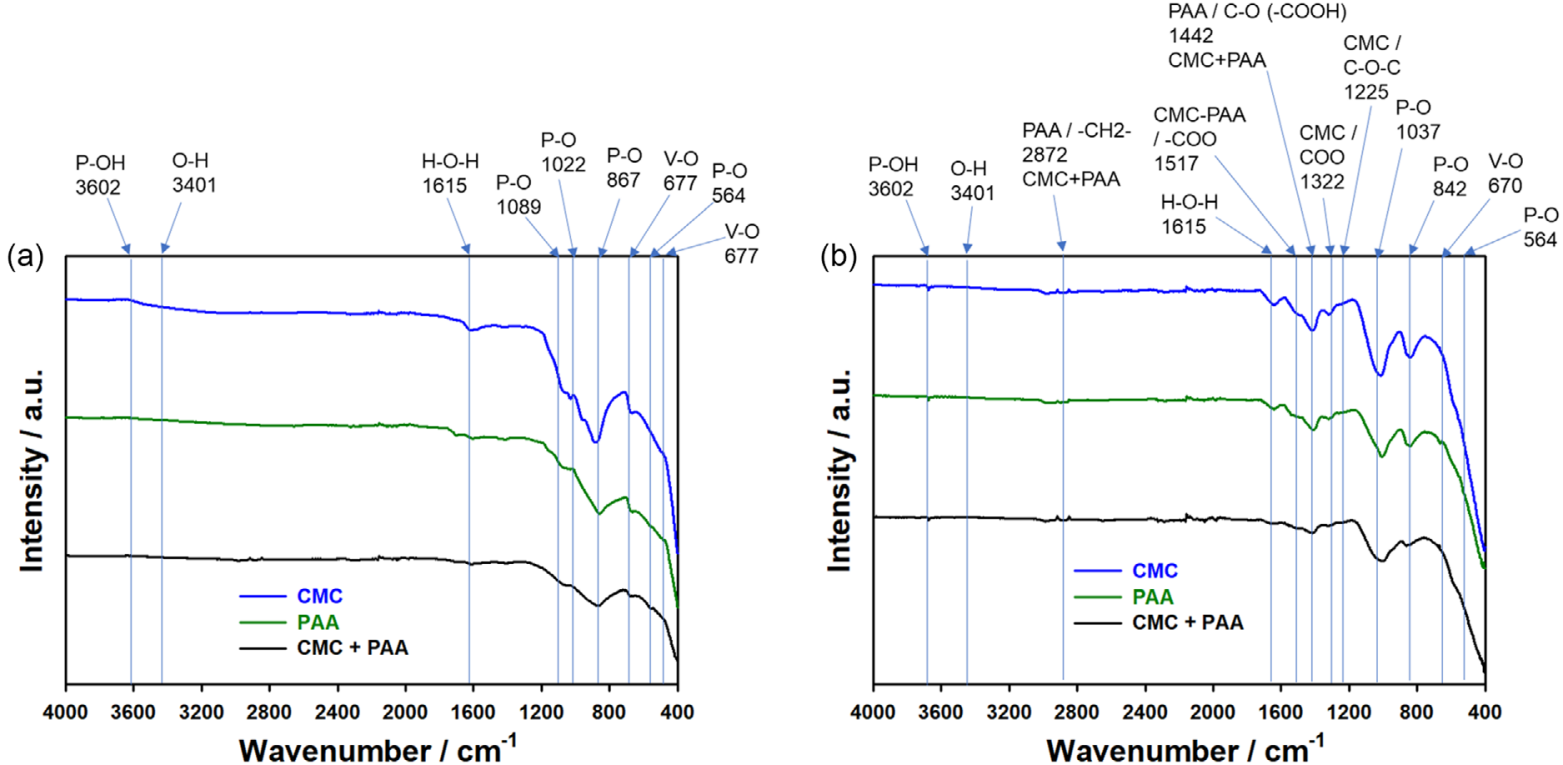
FTIR analysis of VOPO_4_ 2H_2_O surface anodes a) before and b) after cycling in the organic electrolyte of 1 M LiPF_6_ in EC DEC (1:1, v/v) with PVDF, CMC, PAA, and CMC+PAA binders in the potential range of 0.01–2.0 V at 25 °C.

Figure 2a shows the charge/discharge cycling performance of the samples in the potential range of 0.01–2.0 V by applying different current densities. For the same series of samples, we observed the capacity values of 979, 1285, 1411, and 1200 mAh g^−1^ for the lithiation process. The initial delithiation capacities of the VOPO_4_ 2H_2_O samples exhibited a capacity of 460, 425, 528, and 528 mAh g^−1^ for samples made with PVDF, CMC, PAA, and CMC+PAA binders, respectively. The initial capacities for 1st cycle had a largely irreversible capacity loss related to the formation of solid electrolyte interphase (SEI) layers resulting from the decomposition of the electrolyte components and redox couple of V^3+^/V^4+^.^[^ ^10^, ^32^ ^]^ It was also confirmed via the lower Coulombic efficiency (CE) for the 1st cycle is 47.1, 33.1%, 37.4%, and 43.9% for PVDF, CMC, PAA, and CMC+PAA, respectively. However, it should be noted that from the second cycle and subsequent cycles for the samples, the cells exhibited a stable capacity in a range from 170 to 280 mAh g^−1^ after 50 cycles, depending on the samples. This is consistent with the cycling results and voltage curves of the obtained samples, as shown in Figure 2b–d. After 50 cycles, the capacity loss for the PVDF, CMC, PAA, and CMC+PAA is 0.78%, 1.08%, 1.42%, and 0.56% per cycle, while after 100 cycles, the capacity loss is 0.61%, 0.73%, 1.04%, and 0.33% per cycle, respectively. VOPO_4_ 2H_2_O, having an alpha phase with a layered structure, has irreversible changes between the redox couple of V^3+^/V^4+^; however, thanks to its structure stability, this material can maintain stable cycle performance, as shown in Figure 2a. It is important to underline that the sample prepared with aqueous binder samples shows different electrochemical behavior compared to the PVDF sample for the VOPO_4_ 2H_2_O anode, which can be due to different reaction kinetics and position of the redox couple of V^3+^/V^4+^.^[^ ^10^ ^]^ The combination of CMC and PAA as binders appears to be successful in improving the surface stability of the electrode and preventing the electrode from direct contact and attack by the organic components of the electrolyte. The binder system of CMC+PAA can prevent the dissolution of vanadium ions and the formation of side reaction components on the surface of the VOPO_4_ electrode. This indicates that the CMC+PAA binder has a higher structural stability when compared to the PVDF, CMC, and PAA binders (**Figure** **3**). The presence of CMC+PAA in the composite VOPO_4_ electrode was confirmed by SEM images after repeated charge and discharge cycles (**Figure** **4**). Furthermore, SEM images of the CMC‐PAA binder before/after cycling demonstrate the formation of a stable SEI layer, which reduces the dissolution of vanadium ions (as indicated by ICP data) and enhances lithium ion diffusion (coefficient diffusion data) and electronic transport (electronic conductivity data) compared to the other binders (**Figure** 2 and **5**). These improvements collectively enhance the efficiency and capacity of the VOPO4 electrode materials during cycling.

The ICP data show a higher vanadium ion concentration in PVDF (350 mg l^−1^) than in CMC (62 mg l^−1^), PAA (63 mg l^−1^), and CMC+PAA (160 mg l^−1^) after cycling. Therefore, the CMC+PAA binder for the VOPO_4_ electrode can stabilize the structure of the electrode and reduce the dissolution of vanadium ions, effectively reducing the contact between the VOPO_4_ electrode and the components of the organic electrolyte. In addition, the sample with CMC+PAA exhibited a high CE and low irreversible capacity loss for the CMC and PAA samples, thanks to its stable structure and a better reversible exchange reaction between crystal water and lithium‐ion. Furthermore, the obtained results demonstrate that the stable crosslinked structure of CMC+PAA plays an important role in improving the electrochemical performance at various current densities. Thus, we can conclude that the significantly enhanced electrode capacity and stability observed with the CMC‐PAA binder, as compared to solo binders (PVDF, CMC, and PAA), can be attributed to several specific mechanisms. The synergistic interaction between CMC and PAA results in improved mechanical properties and adhesion (Figure S1, Supporting Information, and Figure 4). Specifically, CMC contributes flexibility and mechanical strength, while PAA provides robust adhesion and stability. This mixed binder system facilitates the formation of a stable and uniform SEI on the electrode surface, with PAA playing a crucial role in developing a protective SEI that mitigates electrode degradation (Figure 4). Furthermore, the combination of CMC and PAA enhances ion transport within the electrode, thereby reducing internal resistance and improving overall electrochemical performance (Figure 2 and 3). A similar effect is observed for silicon anodes, where this type of binder can effectively accommodate volume changes during cycling, thereby reducing mechanical stress and preventing electrode cracking.^[^ ^20^, ^22^, ^23^ ^]^

The active electrode materials, especially cathodes, often show a poor electronic conductivity (10^−2^–10^−7^ S cm^−1^), which has an impact on the electrochemical performance for long‐term cycle life.^[^ ^33^, ^34^ ^]^ When a binder is an electrical insulator, the kinetic behavior of lithium transport is naturally negatively influenced. Moreover, the components of cathode materials, such as binders, which are electrical insulators, impact the kinetic behavior, such as lithium transport and power performance. Therefore, the conductive additives of the carbonaceous materials can improve the electronic conductivity, however, optimization of binders and conductive agents is required to reach a higher performance. Usually, the electronic conductivity is determined via various techniques such as the four‐probe method,^[^ ^35^ ^]^ DC polarization,^[^ ^36^ ^]^ and electrochemical impedance spectroscopy (EIS).^[^ ^37^ ^]^ Thus, a deep knowledge of the electrode materials is crucial for understanding the electronic and ionic conductivities of composite electrodes for reaching stable and higher power and cycling performance.

About electric studies, VOPO_4_ electrodes with aqueous‐based binders of CMC, PAA, and CMC+PAA were investigated by DC measurements. Applying a constant DC polarization to the samples with ion‐blocking electrodes, after reaching the equilibrium, the ionic current is totally blocked, and the residual current is only related to the electronic contribution. The electronic conductivities were measured from V/I spectra. These values of electronic conductivities for the samples show that CMC+PAA (1.80 10^−3^ S cm^−1^) has a high value compared to the CMC (1.39 10^−3^ S cm^−1^), PAA (8.17 10^−4^ S cm^−1^), and PVDF (4.56 10^−4^ S cm^−1^) samples in agreement with cycling results and capacities shown in Figure 2. The lower value of electronic conductivities for CMC and PAA leads to a capacity fading for long‐term cycle life than that for the CMC+PAA and PVDF samples.

To understand the electrochemical lithiation/delithiation process of the VOPO_4_ 2H_2_O samples with PVDF, CMC, PAA, and CMC+PAA, the cyclic voltammetry (CV) measurements were performed in the potential range of 0.01–2.1 V at a scan rate of 0.01 mV sec^−1^, as shown in Figure 3a. As mentioned above, the 1st cycle corresponds to the redox couple of V^3+^/V^4+^, which is not reversible after the 1st cycle as discussed in former reports.^[^ ^38^, ^39^ ^]^ The main peak between 1.0 and 1.2 V can be attributed to the formation of the Li_x_VOPO_4_ phase with Li ions inserted into the structure of the VOPO_4_ and a reversible exchange reaction between the Li ions and crystal water occurs during cycling. The oxidation peak observed between 0.6 and 0.7 V is related to the delithiation process and it indicates the reversible exchange reaction processes of Li ions and crystal water, without forming new species during cycling.^[^ ^10^ ^]^ However, it is possible to form various redox couples of V^*x*+^/V^*x*+^ that can occur above 2.0 V, as was reported previously.^[^ ^40^ ^]^ The CV curves of the VOPO_4_ 2H_2_O anodes with aqueous binders show a similar electrochemical behavior to PVDF indicating that the aqueous binders are not actively involved in the lithiation/delithiation processes.

Figure 3b shows the EIS analysis of the VOPO_4_ 2H_2_O with PVDF, CMC, PAA, and CMC+PAA binders. The Nyquist plots consist of a semicircle in the high‐frequency range and a sloping straight line attributed to the low‐frequency range. The plots were fitted via an equivalent circuit having the electrolyte resistance (*R*_e_), capacitance (*CPE*_sei_) of the surface film, resistance (*R*_sei_) of the surface film, double layer capacitance (*CPE*_dl_), charge‐transfer resistance (*R*_ct_), and Warburg (W) impedance related to the diffusion of lithium ions. As shown in Figure 3b, the resistance of the formed SEI layer decreased during cycling, suggesting that the SEI layer plays an important role in the electrode kinetics. Thanks to the additional presence of water, the use of aqueous binders improved the cycling performance of the VOPO_4_ 2H_2_O anode compared to the anode with PVDF binder.^[^ ^10^ ^]^ The resistance (*R*_sei_) of the surface film of PVDF (7.93 and 22.14 Ohm), CMC (53.33 and 42.41 Ohm), PAA (55.76 and 48.46 Ohm), and CMC+PAA (94.79 and 88.18 Ohm) after the 5th and 10th cycles, respectively. The presence of the CMC+PAA binder has a smaller impact on the charge transfer resistance than other binders of PVDF, CMC, and PAA in the electrode. PVDF initially demonstrated the lowest resistance, but this increased substantially by the 10th cycle, suggesting potential degradation. In contrast, CMC and PAA began with higher resistance values, which decreased by the 10th cycle, indicating stabilization of the SEI layer. Although the CMC+PAA binder exhibited the highest initial resistance, the relatively smaller increase between the 5th and 10th cycles points to greater stability and reduced degradation overtime. Moreover, the EIS results demonstrated that the CMC+PAA binder sample shows a more beneficial role in the lithium‐ion, electron transport, and reversible exchange reaction between the lithium ions and crystal water, thanks to a stable structure.

To further validate the influence of aqueous binders on lithium‐ion transport within the electrode, the chemical diffusion coefficient of lithium (DLi) was determined after the fifth cycle using Equation (1).^[^ ^41^ ^]^

(1) $$D_{\text{Li}}=\;0.5\;R^{2}T^{2}A^{-2}F^{-4}C^{-2}u^{-2}\left({\text{cm}}^{2}s^{-2}\right)$$

where *R* is the gas constant, *T* is the temperature, *A* is the electrode area, *F* is the Faraday constant, *C* is the molarity of the lithium‐ion, and *u* is the Warburg term obtained from the slope of angular velocity and Z′ of the electrode. The values of the chemical diffusion coefficients are 7.12 × 10^−16^ cm^2^ s^−2^, 1.04 × 10^−15^ cm^2^ s^−2^, 3.07 × 10^−15^ cm^2^ s^−2^, and 3.97 × 10^−15^ cm^2^ s^−2^, for the VOPO_4_ 2H_2_O with PVDF, CMC, PAA, and CMC+PAA samples, respectively. Among all the samples, the CMC+PAA exhibits a high diffusion coefficient compared to the other binders, showing a good lithium transport during cycling, resulting in its enhanced rate capability, a stable SEI layer on its surface, and better reversible exchange reactions. In addition, the aqueous binders have higher chemical diffusion coefficients compared to the organic binder of PVDF, which is also associated with stable and higher cycling performance.^[^ ^10^ ^]^

**Figure 6:**
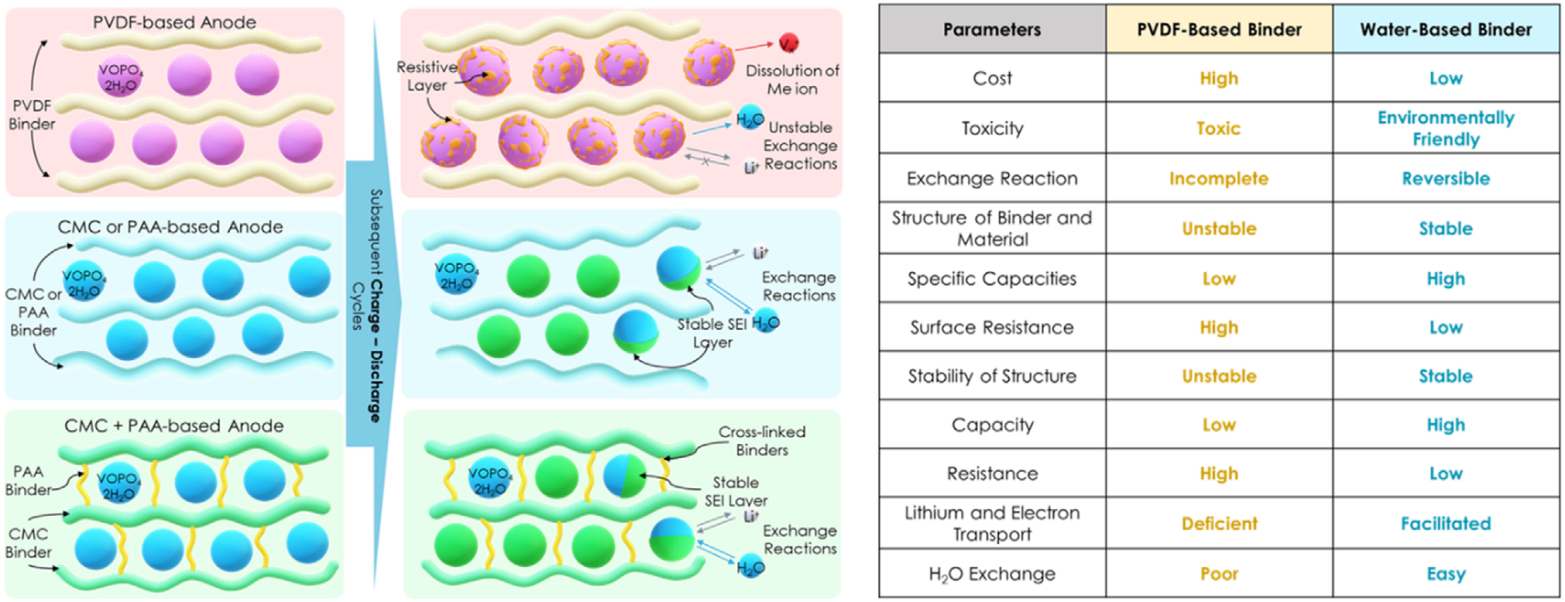
Schematic illustration of the electrochemical exchange reaction between lithium ions and crystal water molecules of VOPO_4_·2H_2_O during subsequent charge–discharge cycles in the organic electrolyte of 1 M LiPF_6_ in EC DEC (1:1, v/v) with organic‐based (PVDF) and aqueous‐based (CMC, PAA, and CMC+PAA) binders and the main advantages of aqueous‐based binder anode over PVDF‐based binder anode are highlighted.

Optical images and SEM tests of the electrodes were performed to investigate the morphology of the VOPO_4_ 2H_2_O samples with PVDF, CMC, PAA, and CMC+PAA binders before versus after cycling, as shown in Figure S1 (Supporting Information) and Figure 4, respectively. The cells were opened after long charge–discharge cycling and were compared to the electrodes before the test. The optical images of the electrodes before and after cycling tests are shown in Figure S1 (Supporting Information). The aqueous binder‐based VOPO_4_ 2H_2_O electrodes were stable and did not undergo any changes after cycling. It means that the electrodes with aqueous binders adhere better on a copper current collector stabilizing the final electrochemical performance (Figure 2). In comparison with the optical images of the electrodes with the aqueous binders before and after cycling tests, it was analyzed the surface morphology of these electrodes via the SEM tests is shown in Figure 4. The SEM images of the electrodes indicate that good adhesion properties of samples with aqueous binders. However, based on the cycling results, the CMC‐PAA sample still has a much better adhesion mechanism than the PVDF, CMC, and PAA samples. We can suppose that the better adhesion properties of the CMC+PAA sample have outstanding strength on the copper collector, thanks to the crosslinked bond formation of the 3D network via the CMC and PAA. By using the combination of the aqueous binder of CMC and PAA, a stronger polymer chain is formed, which has good adhesion to the copper foil and components of the electrodes of the VOPO_4_ 2H_2_O and carbon black (**Figure** **6**). Furthermore, after evaluating the SEM images of the electrodes, the surface of the CMC+PAA sample is more homogenous than the CMC and PAA samples, which indicates that the CMC+PAA binder can prevent the formation of the side reaction components and has better adhesion properties after the cycling test. By contrast, the PVDF, CMC, and PAA samples have shown cracks and holes, justifying the poor electrochemical performance of the CMC+PAA sample (Figure 2). Notably, the PVDF, CMC, and PAA samples have a rough passivation layer on the surface related to the possible formation of the side reaction components, while the CMC+PAA sample looks more stable, as mentioned previously.

The FTIR analysis was carried out on the electrodes before and after cycling, as shown in Figure 5. The stretching vibration related to the P–OH, O–H, and H–O–H has similar positions of peaks for the electrodes before versus after cycling. This result indicates that these groups are attributed to the inner‐surface hydroxyl groups, indicating the hydrous nature of the samples and the presence of crystal water. The positions of the V–O and P–O stretching vibrations refer to the VO_6_ and PO_4_ groups before cycling were changed after cycling (**Table** **2**). It can be related to the impact of the crystal water on the structure and surface morphologies (Figure S1, Supporting Information) and Figure 4). The bands at ≈2870, ≈1520, ≈1440, ≈1320, ≈1225, and ≈1040 cm^−1^ are associated with hydrocarbons present in the samples, and the positions of peaks are changed from samples before and after cycling (Table 2). It should be noted that the band at ≈1615 cm^−1^ for the CMC‐PAA sample related to the crystal water has a similar position and intensity before and after cycling, it can be attributed to the stable content of the water into the structure which has a positive impact on the electrochemical performance compared to the PVDF, CMC, and PAA samples (Figure 2).

**Table 2:** FTIR parameter of the VOPO_4_ 2H_2_O electrodes with PVDF organic binder as reference, CMC, PAA, and CMC‐PAA aqueous binders before and after cycling.

FTIR	Electrodes before cycling			Electrodes after cycling			Powder
Absorption peaks	CMC	PAA	CMC+PAA	CMC	PAA	CMC+PAA	VOPO_4_ 2H_2_O
P—OH	3602	3602	3602	3679	3676	3672	3609
O—H	3401	3401	3401	3401	3401	3401	3401
H—O—H	1614	1606	1615	1640	1639	1652	1615
P—O	1089	1081	1074	1014	1007	1003	1136
P—O	1022	1021	1022	954	951	949	1031
P—O	883	864	868	845	842	853	931
V—O	677	673	675	688	669	675	685
P—O	554	564	556	568	556	572	562
V—O	497	486	489	482	482	482	482
–CH_2_ hydrocarbonates	–	–	–	2896	2881	2882	–
—COO	–	–	–	1513	1509	1501	–
C—O (–COOH)	–	–	–	1423	1416	1416	–
COO	–	–	–	1318	1314	1309	–
C—O—C	–	–	–	1243	1231	1228	–

Based on the physicochemical and electrochemical results discussed above, we can conclude that the aqueous binders for VOPO_4_ 2H_2_O anodes can exhibit an enhanced cycling performance than to the PVDF samples due to the reversible exchange reaction between the crystal water and lithium ions, low surface resistance, and better adhesion properties. Moreover, the low cost and environmental friendly aqueous binders can be attractive for application in lithium‐ion batteries (**Table** **3**).^[^ ^42^ ^]^

**Table 3:** Comparison performance of VOPO_4_‐based materials as anode and cathode for lithium battery systems.

Binder	Electrode	Material	Potential range, V (vs Li/Li^+^)	Capacity in the 1st cycle [mAh g^−1^]	Capacity in the nth cycle [mAh g^−1^]	References
PVDF	anode	VOPO_4_	0.01−2.5 V	1356 (0.1 C)	300 (0.2 C, 100 cycles)	[16]
	anode	LiVOPO_4_	0.01−3.5 V	725.5 (0.1 C)	308.5 (20 mA g^−1^, 30 cycles)	[17]
	anode	VOPO_4_·2H_2_O	0.01−2.0 V	979.1 (0.05 C)	223.6 (0.1 C, 50 cycles	This work
CMC	anode	VOPO_4_·2H_2_O	0.01−2.0 V	1285.4 (0.05 C)	175.7 (0.1 C, 50 cycles	This work
PAA	anode	VOPO_4_·2H_2_O	0.01−2.0 V	1411.7 (0.05 C)	250.6 (0.1 C, 50 cycles	This work
CMC+PAA	anode	VOPO_4_·2H_2_O	0.01−2.0 V	1200.7 (0.05 C)	288.0 (0.1 C, 50 cycles	This work

Figure 6 proposes a schematic illustration showing the electrochemical exchange reaction between lithium ions and crystal water molecules of VOPO_4_·2H_2_O with PVDF organic‐based and CMC, PAA, and CMC+PAA aqueous‐based binders during subsequent charge–discharge cycles. About practical application, the VOPO_4_ 2H_2_O material has been investigated as anode and cathode materials for battery energy storage with the migrating ions of Li, Na, Mg, Zn, and K. Thanks to the structural stability and various structural phases can significantly influence the final electrochemical performance regardless of the migrating ions.^[^ ^17^, ^43^ ^]^ The structure of VOPO_4_ 2H_2_O consists of the VO_6_ and PO_4_ groups, which form a tetragonal 2D slab, structural and crystal water, which is located in the interlayer space.^[^ ^10^ ^]^ Therefore, we can suppose that an exchange reaction between the lithium ions and crystal water is pivotal for enhancing and stabilizing the cycling performance of VOPO_4_ 2H_2_O material. First, during the first discharge process, the lithium ions interact with the VOPO_4_ structure instead of the crystal water extracted from the structure. During the charging process, a complete reverse reaction between the Li ions and crystal water improved the stability of the structure of the VOPO_4_ material with the aqueous‐based binders. This amount of water from the aqueous binder can form a stable SEI layer on the VOPO_4_ surface than that of PVDF, having the resistive layer consisting of the side reaction components formed during the cycling, resulting in structural instability and capacity fading due to the poor ionic and electronic transfer. We can suppose that the resistive layer on the surface of VOPO_4_ with PVDF binder is also attributed to the dissolved vanadium ions, leading to the formation of structural vacancies that could not be filled by the crystal water, unlike the case of the VOPO_4_ sample with the aqueous binder. Even at the lower Coulombic efficiency of the first cycle, the presence of suitable water obtained from the aqueous binders can be affected by the reversible specific capacities and Coulombic efficiency during a long‐term life, the stable surface resistance, and lithium transport compared to the PVDF samples. Additionally, it affects the complete reverse reaction between the lithium ions and crystal water for the charge and discharge cycling. We believe that due to the presence of a suitable amount of water in the VOPO_4_ structure, thanks to the aqueous‐based binder compared to the PVDF organic‐based binder, the aqueous binders can contribute to the stable cycling life, low surface resistance, and enhanced lithium transport kinetics.

Thus, in the development of lithium‐ and metal‐ion, and solid‐state batteries, there is still a need to find new and alternative binders and solvents. These solvent–binder interactions should focus on cost‐effective and efficient solutions that can address the current issues with anode and cathode materials and promote their industrialization across various battery systems.^[^ ^44^, ^45^, ^46^, ^47^, ^48^, ^49^ ^]^

## 3 Conclusions

In summary, the hydrothermal VOPO_4_ 2H_2_O material with the structure of αII was successfully synthesized. The synthesized sample with a nanosheet morphology is tested as the anode material in combination with various types of aqueous‐based binders for lithium‐ion batteries. The nanosheets are found to show stable electrochemical performance for long‐term life at various current densities. The high crystallinity of the materials, together with the nanosheet, is considered to enhance the stability of the materials and morphology of this sample, which can be used as an attractive anode material for battery systems, including the high‐voltage cathode materials such as NCM811 (LiNi_0.8_Co_0.1_Mn_0.1_O_2_). In addition, the VOPO_4_ aqueous‐based electrode maintains the high electrochemical cycle life, thanks to the stable formed SEI layer on the surface of the particle resulting in a) the facilitate lithium‐ion diffusion, b) a low resistance, and c) the reversible and complete exchange reaction between water and lithium compared to the PVDF electrode.

## 4 Experimental Section

4.1

4.1.1

##### Materials Synthesis

The VOPO_4_ 2H_2_O anode material was obtained by a facile hydrothermal method was prepared as previously reported.^[^ ^50^ ^]^ Briefly, for synthesis, V_2_O_5_ powders (Aldrich‐Sigma, 99%) and concentrated H_3_PO_4_ (Aldrich‐Sigma, 99%) were stirred in water, followed by magnetic stirring for 2 h. Then the mixed solution was transferred into a 50 mL Teflon vessel and heated at 100 °C for 8 h in a sealed stainless‐steel. A bright‐yellow product was collected and washed with deionized water and acetone and successively dried at 60 °C for 24 h to obtain the final product.

##### Structural Characterization

The crystalline structure of the obtained VOPO_4_ 2H_2_O anode material was determined by XRD analysis using a PANalytical X’Pert Pro diffractometer in Bragg–Brentano geometry with Cu Kα radiation (45 kV, 40 mA) in a 2*θ* range of 5–80° at a scan rate of 0.03° s^−1^. The polymer cap showed a broad peak around 20°2*θ*, which can be observed in the diffraction patterns of samples.

For the VOPO_4_ 2H_2_O sample was obtained the XRD Crystal Structure Analysis. Deposition Number CCDC 1705670 (https://www.ccdc.cam.ac.uk/structures/Search?Ccdcid=200884&DatabaseToSearch=Published) for VOPO_4_ 2H_2_O, the supplementary crystallographic data for this paper. These data are provided free of charge by the joint Cambridge Crystallographic Data Centre and Fachinformationszentrum Karlsruhe Access Structures service (http://www.ccdc.cam.ac.uk/structures). Crystal data for VOPO_4_ 2H_2_O sample (*M* = 197.94 g mol^−1^), tetragonal, space group P4/nmmz (129), *a *= 6.22199 Å, *b *= 6.22199 Å, *c *= 7.42306 Å, *β *= 90, *V *= 287.37 Å^3^, *Dcalc *= 2.24 g cm^−3^.

The surface morphology of the obtained powder, anode materials before and after cycling, was observed using field emission SEM (ZEISS Supra 40) with EDS.

The FTIR (PerkimElmer UATR Spectrum Two) was detected within a wavenumber interval of 4000–400 cm^−1^ using a scan rate of 8 cm^−1^.

ICP‐OES measurements were performed to determine the V and P concentrations of as‐synthesized VOPO_4_ 2H_2_O powder, and V concentrations in the samples after cycling were measured using an ICP‐OES of PerkinElmer ICP–OES type OPTIMA 7300 DV. The elemental analysis using a Carlo‐Erba EA1108 CHNS‐O was performed to determine the carbon content of the samples.

##### Electrochemical Characterization

VOPO_4_ 2H_2_O‐based electrodes were prepared by blending obtained powder (80 wt%), a polymeric binder (10% w/w, PVDF, CMC (Aldrich, 99%), PAA (Aldrich, 99%), and CMC+PAA (1:1, wt%)) and carbon black (Super C65, Imerys) conductive additive (10 wt%) in 1−1.5 mL of deionized water to form a uniformly mixed slurry via an automatic mixing machine (THINKY MIXER, ARE‐250). The mixtures were cast onto copper foil (15 μm, 99%) with a doctor blade and then dried at 70 °C for 12 h in a convection oven. The PVDF electrode was dried at 120 °C for 10 min to eliminate the NMP solvent. The electrode sheet was then roll‐pressed to ensure interparticle contact. Finally, Circular electrodes of 15 mm and an area of 1.7662 cm^2^ were punched out and redried in a Buchi oven at 80 °C overnight. The active materials mass was around 2.0 mg cm^−2^.

The electrodes were assembled in an argon‐filled glovebox (<0.1 ppm of H_2_O and <0.1 ppm of O_2_) into CR2016 coin‐type cells with VOPO_4_ 2H_2_O anode active material as a working electrode, Li‐metal foil as a counter electrode, polypropylene as the separator (Celgard 2500), and 1 M LiPF_6_ in ethylene carbonate (EC) and diethyl carbonate (DEC) 1/1 v/v (Aldrich) as electrolyte.

The electrochemical tests were performed at 25 °C. The cycling performance was investigated by the MACCOR tester battery cycler in a potential range of 0.01−2.1 V at a current density of 0.1  C. Different C‐rates (0.05, 0.1, 0.2, and 0.5 C) were applied to cells to evaluate C‐rate performance. CV measurements were conducted using the VMP potentiostat (Bio‐Logic) at a scan rate of 0.01 mV s^−1^ in the potential range of 0.01−2.1 V (vs Li^+^/Li). EIS measurement of used active materials in this work was performed on a VMP potentiostat (Bio‐Logic) with the frequency from 100 kHz to 100 mHz at a voltage amplitude of 5 mV. For electrical studies of samples, the casted electrodes were pressed pellets sandwiched between aluminium blocking electrodes with a diameter of 15 mm at a pressure of 1.5 tons cm^−2^ for 3 min using a hydraulic pellet press. The electronic conductivities of samples were tested by linear voltage scanning method (stepwise‐chronoamperometry) at a scanning rate of 0.2 mV s^−1^ from 0 to 500 mV with a step 100 mV using a VMP potentiostat (Bio‐Logic).

## Conflict of Interest

The authors declare no conflict of interest.

## Author Contributions

**Alexander Beutl**: conceptualization, methodology, general experimental investigation and electrochemical analysis, characterization FTIR, XRD, SEM, writing—original draft preparation, writing—original draft preparation. **Andrea Paolella**: characterization FTIR, XRD, SEM, writing—original draft preparation, writing—original draft preparation. **Yuri Surace, Qixiang Jiang, Marcus Jahn**: writing—review and editing. **Artur Tron**: conceptualization, methodology, general experimental investigation and electrochemical analysis, characterization FTIR, XRD, SEM, writing—original draft preparation, writing—original draft preparation.

## Supporting information

Supplementary Material

## Data Availability

The data that support the findings of this study are available from the corresponding author upon reasonable request.
