# Supplementary material for: Aqueous Binders for Electrochemically Stable VOPO4 2H2O Anodes for Li‐Ion Storage
Source: ChemistryOpen. 2025 Jun 17;14(9):e202500102. doi: 10.1002/open.202500102 (PMC12409843; doi:10.1002/open.202500102)
Supplement: Supplementary file 1 — Supplementary Material [file OPEN-14-e202500102-s001.pdf]

Electronic Supporting Information

Aqueous Binders for Electrochemically Stable $VOPO_4\ 2H_2O$ Anodes for Li-Ion Storage

*Alexander Beutl[1], Andrea Paolella[1,2], Yuri Surace[1], Qixiang Jiang[3], Marcus Jahn[1], Artur Tron[1,]**

[1]AIT Austrian Institute of Technology GmbH, Center for Transport Technologies, Battery Technologies, Giefinggasse 2, 1210 Vienna, Austria

[2]Dipartimento di Scienze Chimiche e Geologiche Università degli Studi di Modena e Reggio Emilia Via Campi 103, Modena 41125, Italy

[3]Polymer and Composite Engineering (PaCE) Group, Insitute of Materials Chemistry & Research, Faculty of Chemistry, University of Vienna, Währinger Str. 42, 1090 Vienna, Austria

* Corresponding author: artur.tron@ait.ac.at (Artur Tron)

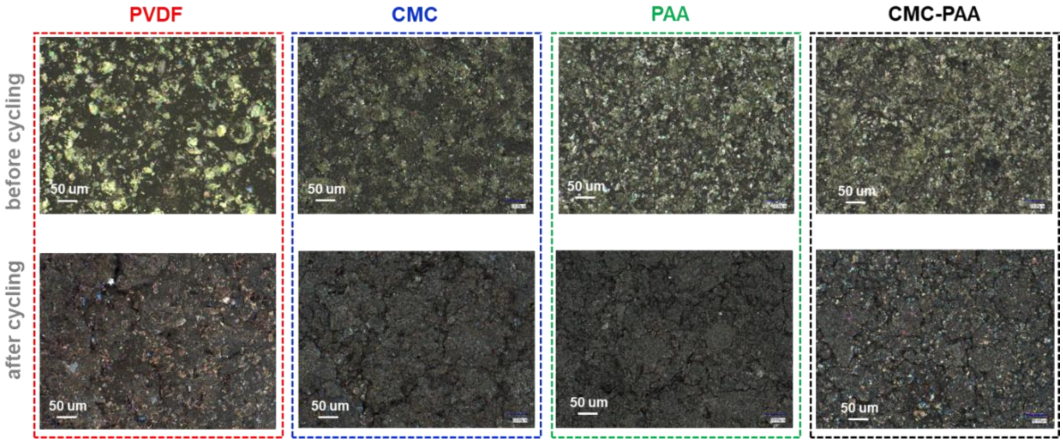

Figure S1. *Surface analysis of $VOPO_4$ $2H_2O$ surface anodes before and after cycling in the organic electrolyte of 1 M $LiPF_6$ in EC DEC (1:1, v/v) with PVDF, CMC, PAA and CMC+PAA binders in the potential range of 0.01-2.0 V at 25°C.*
